# Valorization of camel milk into probiotic-fermented high-energy beverages incorporating Khalas date and nuts: nutritional quality and bioactives profiling

**DOI:** 10.3389/fnut.2026.1843196

**Published:** 2026-05-13

**Authors:** Raed Alayouni

**Affiliations:** Department of Food Science and Human Nutrition, College of Agriculture and Food, Qassim University, Buraydah, Saudi Arabia

**Keywords:** fermented camel milk, food supply, functional food, high-energy beverage, Khalas date, probiotics

## Abstract

**Background:**

Camel milk offers nutritional potential for arid-region functional foods, but high-energy probiotic formulations with balanced macros and bioactives remain underdeveloped.

**Methods:**

This study developed PFHECMs sweetened with 7.5% Khalas date (KD) and fortified with 0% (F1), 15% (F2), or 30% (F3) nut mix to create functional foods with enhanced nutritional and bioactive properties. The formulated beverages were analyzed for proximate composition, phytochemical content, antioxidant activity, color attributes, fatty acid profile, amino acid composition, and volatile compounds using standard analytical procedures.

**Results:**

Proximate analysis revealed dose-dependent improvements: protein tripled from 3.13 g100 mL^−1^ in F1 to 9.13 g100 mL^−1^ in F3, fat increased 5-fold to 14.99 g100 mL^−1^, dietary fiber rose to 1.99 g100 mL^−1^, and energy reached 233 kcal 100 mL^−1^. Phytochemicals surged, with total phenolics climbing from 123.25 mg GAE 100 mL^−1^ in F1 to 506.33 mg GAE 100 mL^−1^ in F3, paralleled by DPPH radical scavenging (173.14–785.29 μmol TE 100 mL^−1^), total flavonoids (17.27–54.29 mg QE 100 mL^−1^), and total flavonols (8.82–22.24 mg QE 100 mL^−1^), confirming strong antioxidant potential. Color became progressively creamier and more appealing, with lightness decreasing from L* 83.56 to 64.66 and yellowness, chroma, and overall color difference increasing. Fatty acid profiles improved markedly, with saturated fats declining from 65.82% in F1 to 32.04% in F3, monounsaturated fats rising to 43.83%, and polyunsaturated fats surging to 24.13% (linoleic acid 21.78%), enhancing the potential of cardiovascular health indices. Amino acid analysis showed nut addition modestly diluted essential amino acids (EAAs) from 27.30% to ≈ 25.81% of total amino acids (TAAs), with EAA: NEAA ratio falling from 0.38 to 0.35, though biological value (54.9–62.4%), essential AA index (88.9–101.3%), and age-group requirement indices remained robust (>50% for adults/schoolchildren). Functional non-essential amino acids (NEAAs), such as arginine, glycine, and cystine, increased, supporting potential metabolic benefits. Volatile GC–MS profiling identified lipid-derived aldehydes (hexanal, nonanal), acids, alcohols, lactones, and nut/date terpenes (caryophyllene, ocimene) as dominant, with F3 exhibiting complex fruity-spicy-nutty aromas versus F1’s pungent dairy notes.

**Conclusion:**

These PFHECMs demonstrate a sustainable use of camel milk by combining camel milk proteins with unsaturated fats, date-derived prebiotic fibers, and nuts to produce high-protein, antioxidant-rich beverages with improved nutritional profiles. While these compositional attributes are consistent with potential benefits for physically active individuals and populations in arid or protein-deficient regions, dedicated *in vivo*, sensory, and consumer studies are required to confirm practical applicability and acceptability.

## 1 Introduction

The growing interest in foods that promote health stems from their ability to help prevent various chronic illnesses and support overall wellness. Nutrient-dense foods rich in bioactive compounds help prevent chronic diseases such as diabetes and cardiovascular disorders while enhancing quality of life (1, 2). Camel milk contains abundant essential nutrients and biologically active components that contribute to its health-supporting properties. Unlike cow’s milk, camel milk is better tolerated by those with lactose intolerance, milk allergies, or diabetes, making it ideal for functional dairy products (3, 4). Emerging research suggests that fermented camel milk may exert protective effects in experimental models of diabetes, hypertension, and inflammatory conditions; however, such findings remain preliminary and context-dependent, and further controlled human studies are needed. Its effectiveness is supported by the presence of bioactive peptides, antioxidant molecules, and beneficial microbes, as evidenced by both experimental and clinical investigations (5, 6). Some experimental studies have reported selective effects of fermented camel milk on dysfunctional or cancerous cells while sparing normal cells, suggesting that it may have biofunctional therapeutic value; however, these observations require corroboration and translation into clinical settings before any therapeutic role can be established (7). Probiotics in fermented camel milk enhance gut microbiota diversity, supporting digestion, metabolism, and immunity, while lactoferrin and immunoglobulins provide strong antimicrobial and antiviral effects (5).

Recent studies have expanded the technological and functional value of camel milk products. *Moringa oleifera* seed extract has been reported as an effective alternative coagulant for the production of fresh camel cheese (8). Starter culture selection also affects proteolysis and the generation of bioactive peptides during camel cheese ripening (9). In addition, blending camel milk with chickpea milk can improve nutritional quality, antioxidant potential, probiotic viability, and sensory acceptability of fermented products. For quality control, Fourier transform mid-infrared spectroscopy combined with chemometrics has shown promise for detecting camel milk adulteration (10).

Adding nuts such as almonds, walnuts, and cashews to fermented camel milk increases protein content and overcomes the bioavailability limitations of plant-based fortifiers (11). Fermented camel milk has superior whey proteins with higher *α*-helix content (32.23%) and no *β*-lactoglobulin, making it hypoallergenic for sensitive groups (12). Nut supplements in fermented camel milk promote protein hydrolysis via convergent protease action from LAB consortia, especially *Lactobacillus helveticus*, *L. bulgaricus*, and *Streptococcus thermophilus* (13). Camel milk fermented with these starters shows greater casein breakdown and higher free amino groups than bovine milk, producing bioactive Val-Pro peptides with ACE-inhibitory activity (13). Nut protein matrices, rich in essential amino acids like lysine, leucine, phenylalanine, arginine, and isoleucine, undergo LAB-driven proteolysis in fermented camel milk to yield a superior combined amino acid profile (14–16).

The functional synergy of fermented nut-supplemented camel milk lies in the reciprocal improvement of digestibility and metabolic accessibility through microbial enzymatic action (17). Recent studies show LAB, such as *Lactiplantibacillus plantarum* and *Limosilactobacillus fermentum*, degrade anti-nutrients (phytates, phenolics, inhibitors), boosting mineral and amino acid bioavailability (18). *L. plantarum* fermentation of camel milk with walnuts boosts protein breakdown, generating new amino acids (serine, leucine, lysine) that increase with fermentation time (19, 20). Camel milk’s thermal stability (32%–35% whey denaturation at 80 °C vs. 70%–75% for bovine) preserves bioactive peptides during nut fortification, making fermented nut-supplemented camel milk ideal for protein support and therapeutic diets (21, 22).

Dates, as natural sweeteners and bioactive sources in fresh/fermented camel milk, enhance the nutritional and health benefits of both (23). Adding 10–30% date material markedly boosts total solids, sugars, minerals, and phenolic antioxidants in camel milk. At the same time, its prebiotic fibers support higher growth and activity of key starter probiotics such as *L. acidophilus*, *B. bifidum*, and *S. thermophilus* (23, 24). Date-fortified camel milk is homogenized with stabilizer, heated to 85 °C, cooled, inoculated with ABT-5, and fermented at 39 °C; 10%–20% date addition optimizes texture, flavor, and function (21, 25). Date-enriched fermented camel milk sustains higher probiotic counts in storage, boosts phenolic antioxidants, and elevates key minerals (K, Mg, Fe, Zn) vs. plain milk (23, 26). Unfermented date-camel milk, processed gently (thermal/non-thermal), retains antimicrobial/bioactive peptides like fermented versions for shelf-stable drinks, though *in vivo*/clinical confirmation of oxidative stress or metabolic benefits is needed (21, 27). Probiotic LAB fermentation of CM releases bioactive peptides and metabolites with antioxidant, free radical-scavenging, metal-chelating, and antimicrobial effects, enhancing gut health, safety, and functionality (28, 29). Probiotic viability during processing and storage is key to achieving health benefits; camel milk outperforms cow’s milk in protecting probiotics during digestion, with skim milk and trehalose enhancing freeze-drying survival (24, 30). Bacteria survive stresses through stress protein synthesis, compatible solute accumulation, and cross-protection strategies that enhance resilience during processing (31).

This study aims to develop a novel probiotic-fermented, high-energy camel milk beverage using date powder as a natural sweetener and prebiotic fiber source. Specifically, it seeks to create an innovative formulation that combines nut protein supplementation with date powder, fermented using selected probiotic lactic acid bacteria (LAB) strains, and then to evaluate its nutritional profile, physicochemical characteristics, phytochemical and antioxidant properties, amino and fatty acid composition, and volatile profile. The resulting compositional data are intended to support the future design of functional beverages, while recognizing that confirmation of health effects will require dedicated in vivo and clinical investigations.

## 2 Materials and methods

### 2.1 Materials

Raw camel milk from Wadah breed camels (5–9 years old) was obtained in the morning from the College of Agriculture and Food farm at Qassim University, Qassim region, Saudi Arabia, from September to December 2025. Proximate composition analysis of 100 mL raw milk revealed 87.7 g water, 3.27 g protein, 3.02 g fat, 8.86 g solids non-fat, 4.78 g lactose, and 0.81 g ash (32). These raw camel milk composition values, obtained in a previous study under comparable conditions, were used as a reference to contextualize the magnitude of the nutritional changes induced by date and nut fortification; however, raw, unfortified milk was not included as a separate experimental treatment in the present trial. The KDs (Phoenix dactylifera L.), harvested in 2025, were purchased from Nakheel Aldka LTD, Riyadh, Saudi Arabia. Per 100 g fresh pitted dates: 15.48 g water, 2.71 g protein, 0.39 g fat, 2.89 g ash, 78.53 g total carbohydrates, 7.24 g dietary fiber. Direct Vat Set (DVS) ABT-5 culture (*Lactobacillus acidophilus* LA-5, *Bifidobacterium bifidum* BB-12, *S. thermophilus*) was sourced from Chr. Hansen (Copenhagen, Denmark) via Misr Food Additives (MIFAD), Badr City, Egypt. Simultaneously, nuts were procured from Damascus Winds supermarket in Buraydah, Qassim Region, Saudi Arabia. For each 100 g nut blend, the following were combined in a knife blender (Santos, VM0122E, Cleveland, OH, United States) at speed 3 for 60 s minimum to yield a uniform mixture: 20 g roasted almonds, 20 g roasted cashews, 20 g roasted peanuts 20 g walnuts, 5 g pistachios, 5 g hulled pumpkin seeds, 5 g hulled sunflower seeds, 5 g chia seeds. The resulting nut mix was stored at 4 ± 1 °C before subsequent processing.

### 2.2 High-energy sweetened camel Milk preparation

The KDs were carefully washed, air-dried, and homogenized to create a consistent KD paste (KDP). Fresh camel milk (6 L total) was collected post-milking and transported under cooling conditions to the lab within 1 h. It was then split into three 2-L batches for processing as follows. Formula 1 (F1) consisted of camel milk incorporated with 7.5% Khalas date paste (KDP) and served as the formulation control representing a date-sweetened but nut-free high-energy beverage. Formula 2 (F2) and Formula 3 (F3) were prepared by incorporating 7.5% KDP together with 15 and 30% (w/v) of the nut mix, respectively, to provide incremental increases in protein, fat, and phytochemical content. The choice of 7.5% KDP was based on previous work indicating that 7–10% date addition yields acceptable sweetness and texture in fermented camel milk. In comparison, the 15 and 30% nut inclusion levels were selected to approximately double and triple the protein content of plain camel milk, respectively, without excessively increasing viscosity or phase separation, as observed in preliminary trials and in the literature on high-protein dairy beverages. The nut mix was added to raise the protein content to more than 6% in F2 and 9% in F3, effectively doubling and tripling the protein level, respectively, compared with plain camel milk. Commercial high-protein milk drinks commonly use protein levels of 5–10 g 100 mL^−1^ (10–20 g per 200 mL serving) to meet “high protein” thresholds (33, 34). All formulas were heated with stirring to 40–50 °C, held 10 min, homogenized in a knife blender (Santos, VM0122E, Cleveland, OH, United States) at speed 6 for 120 s, then filtered through 2-layer cheesecloth. All filtered batches were pasteurized (75 °C for 5 min), cooled to 40 °C, and then inoculated.

### 2.3 Preparation of PFHECMs

Following cooling to 40 °C, F1, F2, and F3 were each inoculated with 0.75 g L of the DVS ABT-5 starter culture (containing *L. acidophilus* LA-5, *B. bifidum* BB-12, and *S. thermophilus*), thoroughly mixed to ensure uniform distribution, and incubated at 42 °C for 6–8 h until pH reached 4.5–4.7. The pH of several samples was measured using a portable digital pH meter (HANNA HI 8314, Romania). Later, the fermented milks were transferred to a cooling environment at 4 ± 1 °C before subsequent analyses.

### 2.4 Nutritional composition of PFHECMs

All formulations were focused on detailed proximate analyses, including moisture (AOAC 925.10), crude protein (AOAC 981.10), fat ((35).18, Gerber butyrometer), ash (AOAC 942.05), total dietary fiber (AOAC 992.16), available carbohydrates (by difference), and energy content, per standard AOAC protocols (2000). Analyses were performed in triplicate with calibration standards and quality controls incorporated in each batch.

### 2.5 Phytochemical analysis of PFHECMs

Total phenolic content (TPC) of the PFHECM was examined by the Folin–Ciocalteu assay, and results were expressed as milligrams of gallic acid equivalents per 100 mL (mg GAE 100 mL^−1^), following Bettaieb *et al.* (36). Antioxidant activity was evaluated by the DPPH radical scavenging assay (DPPH-RSA), in which the decrease in absorbance of the 2,2-diphenyl-1-picrylhydrazyl solution was used to calculate the percentage inhibition. Values were expressed as micromoles of Trolox equivalents per 100 mL (μmol TE 100 mL^−1^) based on a Trolox calibration curve (37). In addition, total flavonoids (TF) were quantified according to the procedures of Barakat and Almundarij (38) with results expressed as milligrams of quercetin equivalents per 100 mL (mg QE 100 mL^−1^). Total flavonol (TFL) contents were determined using the method of Kumaran & Karunakaran (39), with results expressed as milligrams of quercetin equivalents per 100 mL (mg QE 100 mL^−1^).

### 2.6 Instrumental color measurements of PFHECMs

The color attributes of fresh PFHECM samples were determined using a HunterLab ColorFlex colorimeter (Reston, VA, United States). Before measurement, the instrument was calibrated using a standard white calibration tile according to the manufacturer’s instructions. Each PFHECM sample was measured under the black measurement cup of the Hunter Lab colorimeter to ensure consistent light shielding and minimize external light interference during color assessment. Color was expressed in the CIE Lab. color, where L* represents lightness, a* indicates the red-green axis, and b* corresponds to the yellow-blue axis. The hue angle (H°), chroma (C), and color changes (∆E) were then calculated relative to the control according to Lavelli et al. (40).

### 2.7 Determination of the FAs profile in PFHECM

Total fatty acids (FAs) in PFHECMs were converted to fatty acid methyl esters (FAMEs) as described by Aldai et al. (41). These FA methyl esters (FAMEs) were separated by gas–liquid chromatography (GLC) coupled to a flame ionization detector (FID), using a programmed temperature gradient: an initial 100 °C ramp to 200 °C, then 2 °C min^−1^ to 230 °C, held for 10 min. Injector and detector temperatures were 250 °C and 300 °C, respectively. Peak integration and data processing were performed with Saturn GC Workstation Software v. 5.51.

### 2.8 Determination of AAs profile in PFHECMs

The amino acid (AA) profiles of the PFHECMs were determined using a Sykam AA analyzer (Sykam GmbH, Germany), equipped with a quaternary pump (S 2100), autosampler (S 5200), reaction module (S 4300) with dual-filter photometer (440–570 nm), and refrigerated reagent organizer (S 4130). Separation was achieved on an LCA K06/Na column under gradient elution with Buffer A (11.8 g Na₃C₆H₅O₇2H₂O, 6 g citric acid, 65 mL methanol, 6.5 mL 32% HCl, 0.5 g phenol L^−1^; pH 3.45, 0.12 N) and Buffer B (19.6 g Na₃C₆H₅O₇2H₂O, 3.1 g NaOH, 5 g boric acid L^−1^; pH 10.85, 0.20 N), at 1 mL min^−1^ and column temperature gradient of 57–74 °C. Detection occurred at 440 and 570 nm. Standards (Sykam Type H, 2.5 μmol m L^−1^ each of 18 AAs, cystine 1.25 μmol m L^−1^) were diluted, filtered (0.22 μm), and injected (100 μL). Samples (~1 g, accurate to 0.0001 g) were hydrolyzed in 6 N HCl (20 mL) at 110 °C for 16 h, filtered, evaporated, reconstituted in 100 mL HPLC water, diluted 1:10, filtered, and injected (100 μL). AA concentrations were quantified via relative response factors from standards (42). The biological value and AA scores were computed according to the WHO (43) guidelines, as detailed by Chavan et al. (44).

### 2.9 Quantification of volatile components in PFHECMs

The volatile components of the PFHECM*s* were analyzed using a Trace GC-TSQ mass spectrometer (Thermo Scientific, Austin, TX, United States) fitted with a TG–5MS capillary column (30 m × 0.25 mm i.d., 0.25 μm film thickness). The oven temperature program began at 50 °C, ramped at 5 °C min^−1^ to 250 °C (hold 2 min), then increased at 30 °C min^−1^ to 300 °C (hold 2 min). Injector and MS transfer line temperatures were set to 270 °C and 260 °C, respectively; helium was used as the carrier gas at 1 mL min^−1^. Samples (1 μL, diluted) were auto-injected in split mode (Autosampler AS1300); solvent delay was 4 min. EI spectra were acquired at 70 eV (m/z 50–650 full scan), with the ion source at 200 °C. Compounds were identified by matching mass spectra against WILEY 09 and NIST 14 libraries (45).

#### 2.9.1 Methylation method

To 10 mg of pre-weighed sample in a vial, 2% (v/v) H₂SO₄ in methanol was added for methylation to fatty acid methyl esters (FAMEs), following Hewavitharana et al., (46). The vial was incubated at 80 °C with intermittent shaking. After cooling, 0.25 mL of 1 M NaOH (neutralized aqueous solution) was added, and the mixture was gently mixed. Then, 1 mL of 5.964% (w/v) KCl was added with vigorous shaking, followed by 1 mL hexane (also with vigorous shaking). The hexane (organic) upper layer was isolated for subsequent GC–MS analysis, following the cited protocol (46).

### 2.10 Statistical analysis

Statistical analysis employed a one-way ANOVA in SPSS v.22 (IBM Corp., Armonk, NY, United States; 2013) under a completely randomized design, with Tukey’s multiple-range test for post-hoc comparisons at *p* < 0.05, as described by Steele (47). The experimental design, therefore, consisted of three formulations (F1, F2, and F3), all fermented under identical conditions, with F1 serving as the formulation control and F2–F3 representing increasing levels of nut fortification; no non-fermented or unsweetened camel milk control was included.

## 3 Results

### 3.1 Proximate chemical composition of formulated PFHECMs

Table 1 shows a certain enhancement in nutritional quality across the three PHPCM formulas (F1, F2, and F3), with all constituents differing significantly (*p* < 0.05). This trend guides the stepped-up fortification strategy: moisture drops sharply from 82.83% in F1 to 69.95% in F2, then to 57.11% in F3. Protein levels triple from a baseline of 3.13 g 100 mL^−1^ in F1 to 6.12 g 100 mL^−1^ in F2 and 9.13 g 100 mL^−1^ in F3, representing roughly 2-fold and 3-fold, respectively. Fat content increased more than 5-fold (from 2.89 to 14.99 g 100 mL^−1^), attributable to nut-derived lipids, alongside proportional fiber increases (0.39 to 1.99 g 100 mL^−1^). Ash and carbohydrates show moderate increases as well, pushing energy density up threefold from 78.13 kcal 100 mL^−1^ in F1 to a robust 233.01 kcal 100 mL^−1^ in F3.

**Table 1:** Proximate chemical composition of different formulated PFHECMs (mean ± SE), *n* = 3.

Composition*	PFHECM formulas		
	F1	F2	F3
Moisture	82.83 ± 0.17^a^	69.95 ± 0.13^b^	57.11 ± 0.11^c^
Protein	3.13 ± 0.11^a^	6.12 ± 0.07^b^	9.13 ± 0.08^c^
Total fat	2.89 ± 0.04^a^	8.93 ± 0.02^b^	14.99 ± 0.01^c^
Ash	0.84 ± 0.06^a^	1.15 ± 0.08^b^	1.37 ± 0.01^c^
Dietary fiber	0.39 ± 0.01^a^	1.18 ± 0.03^b^	1.99 ± 0.09^c^
Total carbohydrates	9.91 ± 0.12^a^	12.66 ± 0.05^b^	15.41 ± 0.04^c^
Energy (kcal)	78.13 ± 1.37^a^	155.45 ± 0.29^b^	233.01 ± 0.36^c^

*Presented on 100 mL wet weight, SE: Standard error, ^a,b,c^: F1: fermented camel milk contains 7.5% KDP, F2: fermented camel milk with 7.5% KDP and 15% of Nut mix, and F3: fermented camel milk with 7.5% KDP and 30% of Nut mix. ^a,b,c,^: No significant difference (*p* > 0.05) between any two means within the same row with the same superscripted letters.

### 3.2 Phytochemicals content in formulated PFHECMs

Table 2 demonstrates a compelling dose-dependent surge in phytochemical content and antioxidant capacity across the PHPCM formulas, with all parameters showing highly significant differences (*p* < 0.05). The TPC increases from 123.25 mg 100 mL^−1^ in F1 to 506.33 mg 100 mL^−1^ in F3, reflecting the presence of valuable content in fortified CM. The DPPH-RSA corresponded to the progression of TPC and provided 173.14 to 785.29 μmol TE 100 mL^−1^, confirming a strong correlation between phenolics and bioactivity. In the same context, TF and TFL rise equivalently: TF from 17.27 to 54.29 mg QE 100 mL^−1^, TFL from 8.82 to 22.24 mg QE 100 mL^−1^. These results highlight F3 as a potent antioxidant-rich functional beverage, far exceeding plain camel milk baselines and entering ideal high-phenolic territory.

**Table 2:** Phytochemical contents of different formulated PFHECMs (mean ± SE), *n* = 3.

Phytochemicals*	PFHECM formulas		
	F1	F2	F3
TPC [mg GAE 100 mL^−1^]	123.25 ± 6.13^c^	311.24 ± 9.53^b^	506.33 ± 10.74^a^
DPPH-RSA [μmol of TE 100 mL^−1^]	173.14 ± 5.85^c^	458.73 ± 10.47^b^	785.29 ± 8.37^a^
TF [mg QE 100 mL^−1^]	17.27 ± 2.32^c^	30.95 ± 4.28^b^	54.29 ± 5.58^a^
TFL [mg QE 100 mL^−1^]	8.82 ± 1.98^c^	13.87 ± 2.83^b^	22.24 ± 3.67^a^

^*^Presented on 100 mL wet weight, SE: Standard error, ^a,b,c^: F1: fermented camel milk contains 7.5% KDP, F2: fermented camel milk with 7.5% KDP and 15% of Nut mix, and F3: fermented camel milk with 7.5% KDP and 30% of Nut mix. ^a,b,c,^: No significant difference (*p* > 0.05) between any two means within the same row with the same superscripted letters.

### 3.3 Instrumental color of formulated PFHECMs

Table 3 shows a clear, statistically significant shift in the color properties of the PHPCM formulas as the nut mix level increases. Lightness (L*) drops from 83.56 in F1 (7.5% KDP only) to 67.27 in F2 and 64.66 in F3, indicating a progressive darkening of the product with fortification. The a* coordinate (green–red axis) remains negative in all samples, showing that the drinks stay within the greenish quadrant, but F1 is the least green (−0.53), while F2 and F3 are more negative (−1.08 and −2.42, respectively). The b* coordinate (blue–yellow axis) increases markedly from 5.17 in F1 to 15.26 in F2, then decreases slightly to 11.87 in F3, indicating that F2 is the most yellow and visually the warmest in tone. This pattern is reflected in chroma (C*), which rises from 5.20 in F1 to 15.30 in F2, then to 12.12 in F3, confirming stronger color saturation in the nut-date-fortified formulations, especially F2. Hue angle (H°) persists in the negative range, with F2 and F1 close in value (around −84 to −86°) and F3 somewhat less negative (−78.56°), implying subtle hue shifts likely driven by nut pigments. The total color difference (ΔE) between F1 and the fortified beverages is high (≈19–20). Taken together, these results indicate that adding nut mix on top of 7.5% KDP significantly darkens the product, enhances yellowness and color intensity, and produces visually distinct formulations, with F2 showing the strongest yellow chroma and F3 presenting a slightly darker, less vivid but still strongly colored appearance.

**Table 3:** Instrumental and visual color analysis of different formulated PFHECMs (mean±SE), *n* = 3.

PFHECMs formulas	Visual color parameters					
	L*	a*	b*	C	H°	∆E
F1	83.56 ± 0.58 ^a^	−0.53 ± 0.18 ^b^	5.17 ± 0.54 ^c^	5.20 ± 0.53 ^c^	−84.13 ± 2.32 ^b^	-
F2	67.27 ± 0.43 ^b^	−1.08 ± 0.23 ^a^	15.26 ± 0.34 ^a^	15.30 ± 0.32 ^a^	−85.97 ± 0.90 ^b^	19.07 ± 0.55 ^a^
F3	64.66 ± 0.24 ^c^	−2.42 ± 0.61 ^b^	11.87 ± 0.60 ^b^	12.12 ± 0.66 ^b^	−78.56 ± 2.64 ^a^	19.80 ± 0.22 ^a^

L*, a*, and b* were obtained directly from the Hunter instrument. C, H°, and ∆E were calculated according to the equation indicated in the methods section. F1: fermented camel milk contains 7.5% KDP, F2: fermented camel milk with 7.5% KDP and 15% of Nut mix, and F3: fermented camel milk with 7.5% KDP and 30% of Nut mix, ^a,b,c^: no significant difference (*p* > 0.05) exists between any two means within the row with the same superscripted letters.

### 3.4 Fatty acids profiling of formulated PFHECMs

Table 4 summarize the fatty acid (FA) profiles of the three PFHECMs (F1, F2, and F3) which reveal a progressive shift toward a healthier lipid composition, with total saturated FA (SFA) decreasing from 65.82% (F1) to 49.44% (F2) and 32.04% (F3), monounsaturated FA (MUFA) rising from 32.4 to 37.48 and 43.83%, and polyunsaturated FA (PUFA) surging from 1.77 to 12.86 and 24.13%, respectively. Palmitic acid (C16:0) dominated SFAs in all samples (35.39, 27.08, 18.24%), followed by myristic (C14:0) and stearic (C18:0) acids, while oleic acid (C18:1, 21.59–39.22%) led MUFAs, and linoleic acid (C18:2 n-6) drove PUFA increases (1.49–21.78%). This trend enables successful modulation through formulation changes (e.g., nut/date additions, probiotic fermentation), lowering SFA dominance typical of camel milk while boosting essential PUFAs, thereby enhancing nutritional value for cardiovascular health and functional food applications. Key findings include F3’s more unsaturated FA profile (MUFA+PUFA = 67.96%), low SFA, and raised linoleic/linolenic acids, suggesting optimal lipid quality for a high-energy product; minor FAs like lauric (C12:0) and behenic (C22:0) remained low (<1%), with no short-chain FAs detected, positioning with camel milk’s long-chain bias but improved by ingredients. These profiles verify PFHECMs as healthier alternatives to bovine dairy, with declining SFA/PUFA ratios that potentially lower atherogenic risk.

**Table 4:** Fatty acids profile analysis of different formulated PFHECMs.

Fatty acids*	RT	Formula	F1	F2	F3
Saturated fatty acids					
Lauric acid	15.162	C₁₂H₂₄O₂	0.51	0.62	0.27
Myristic acid	20.572	C₁₄H₂₈O₂	13.2	9.73	5.02
Pentadecanoic acid	23.472	C₁₅H₃₀O₂	1.35	0.98	0.5
Palmitic acid	26.232	C₁₆H₃₂O₂	35.39	27.08	18.24
Margaric acid	29.015	C₁₇H₃₄O₂	2.07	0.77	0.39
Stearic acid	31.622	C₁₈H₃₆O₂	12.78	9.79	7.2
Arachidic acid	36.696	C₂₀H₄₀O₂	0.43	0.36	0.3
Behenic acid	41.477	C₂₂H₄₄O₂	0.09	0.11	0.12
Total			65.82	49.44	32.04
Monounsaturated fatty acids					
Myristoleic acid	22.322	C₁₄H₂₆O₂	0.88	0.64	0.28
cis-10-pentadecenoic acid	24.956	C₁₅H₂₈O₂	0.38	0.27	0.34
Palmitoleic acid	27.392	C₁₆H₃₀O₂	8.38	6.69	3.52
cis-10-Heptadecenoic acid	29.99	C₁₇H₃₂O₂	0.34	0.38	0.21
Oleic acid	32.438	C₁₈H₃₄O₂	21.59	29.43	39.22
cis-11-Eicosenoic acid	37.427	C₂₀H₃₈O₂	0.83	0.07	0.26
Total			32.4	37.48	43.83
Polyunsaturated fatty acids					
Linoleic acid	34.188	C₁₈H₃₂O₂	1.49	11.53	21.78
Linolenic acid	36.347	C₁₈H₃₀O₂	0.28	1.33	2.35
Total			1.77	12.86	24.13

*Presented means are the average of duplicate analyses.

### 3.5 Amino acids profiling of formulated PFHECMs

Table 5 demonstrates the AAs profile in formulated PFHECMs. The addition of nuts to fermented camel milk resulted in a modest shift in the AA profile, with a slight decrease in the proportion of EAAs relative to TAAs and an accompanying increase in NEAAs. Specifically, the EAA content decreased from 27.30% of TAAs in F1 to 25.83% in F2 and 25.81% in F3. This corresponded to an elevation in NEAAs from 72.70% in F1 to 74.17% in F2 and 74.19% in F3, yielding a reduction in the EAA: NEAA ratio from 0.38 in F1 to approximately 0.35 in both nut-supplemented formulas. Individual EAA concentrations reflected this dilution effect, particularly from F1 to F2, where histidine, threonine, methionine, valine, lysine, isoleucine, leucine, and phenylalanine all declined notably per g of protein, with threonine showing the most pronounced reduction (from 40.15 to 31.40 mg g^−1^). Cystine, however, increased substantially (1.25 to 14.29 mg g^−1^), underlining the contribution of sulfur-containing amino acids from nuts. From F2 to F3, several EAAs incompletely recovered (e.g., histidine from 28.06 to 32.21 mg g^−1^, lysine from 35.50 to 39.10 mg g^−1^). However, total EAA levels remained below F1, indicating that the essential compositional shift occurred at the 15% nut level with minimal further alteration at 30%. However, among NEAAs, nut integration drove increases in arginine (50.90 to 64.24 mg g^−1^ across F1–F3), aspartic acid (75.52 to 67.97 and 78.89 mg g^−1^), glutamic acid (169.43 to 170.85 mg g^−1^), glycine (14.38 to 23.43 mg g^−1^), and tyrosine (15.26 to 6.11 mg g^−1^), which largely surpassed F1 values by F3. Proline, a dominant milk-derived NEAA, declined initially (355.68 to 318.31 mg g^−1^) but stabilized, consistent with progressive substitution of casein-rich milk proteins by nut proteins higher in arginine and glutamic acid. Nut addition subtly dilutes camel milk’s EAA density (lysine, leucine, threonine, methionine) while enriching NEAAs (arginine and cystine); 15% nuts optimize this shift without further EAA compromise, supporting a balanced functional food.

**Table 5:** Amino acids profile of different formulated PFHECMs.

AAs*	F1	F2	F3
EAAs mg g protein^−1^			
Histidine	36.16	28.06	32.21
Threonine	40.15	31.40	34.38
Methionine	13.11	6.70	9.02
Valine	36.31	30.40	33.14
Cystine	1.25	14.29	10.23
Lysine	43.20	35.50	39.10
Isoleucine	19.27	16.47	17.62
Leucine	44.11	36.49	40.05
Phenylalanine	37.44	30.56	36.43
Total EAAs	270.99	229.87	252.18
NEAAs mg g protein^−1^			
Serine	27.16	25.63	25.54
Arginine	50.90	50.57	64.24
Asparatic acid	75.52	67.97	78.89
Glycine	14.38	16.75	23.43
Glutamic acid	169.43	155.60	170.85
Alanine	13.22	12.66	15.40
Proline	355.68	318.31	330.49
Tyrosine	15.26	12.49	16.11
Total NEAAs	721.56	659.99	724.95
EAAs % of TAAs	27.30	25.83	25.81
NEAAs % of TAAs	72.70	74.17	74.19
EAAs: NEAAs ratio	0.38	0.35	0.35

*Presented means are the average of duplicate analyses.

Table 6 summarizes key amino acid groups and derived protein quality indices for PFHECMs, expressed as mg g^−1^ protein alongside BV, EAAI, and requirement indices for different age groups. Notable findings include high total BCAAs at 99.69, 83.35, and 90.81 mg g^−1^ across F1-F3, representing 37% of total EAAs and supporting muscle anabolism. Total basic AAs were 130.26, 114.13, and 135.54 mg g^−1^, with F3 exceeding F1 due to arginine enrichment from nuts. Aromatic AAs, uncharged polar AAs, and conditional AAs showed modest variations: aromatic dipped to 43.05 mg g^−1^ in F2 before recovering to 52.54 mg g^−1^ in F3; uncharged polar AAs remained stable at 83–86 mg g^−1^; conditional AAs were highest in F3, 476.71 mg g^−1^. Protein quality metrics reveal robust nutritional adequacy: BV ranged 54.9–62.4% (F2 lowest, F1 highest), EAAI 88.9–101.3% (F1 > 100% indicating superior preschool reference match), and requirement indices >50% for preschool/schoolchild/adult across all samples, though lower for infants (34.2–38.3%). Sulfur and aromatic AAs were limiting, typical for plant-enriched dairy. In the broader context, these profiles confirm that nut supplementation maintains camel milk’s high-quality protein (EAA ~ 26% TAAs) while boosting functional groups such as BCAAs/basic AAs and conditional AAs, positioning formulations as sustainable functional foods.

**Table 6:** The AAs% and calculated biological efficiency, essential AA index, estimated protein efficiency ratio, and requirement index of different age groups.

Parameters	F1	F2	F3
Total BCAAs (mg g^−1^ protein)	99.69	83.35	90.81
Total BAAs (mg g^−1^ protein)	130.26	114.13	135.54
Total Aromatic AA (mg g^−1^ protein)	52.70	43.05	52.54
Total uncharged polar AAs (mg g^−1^ protein)	83.81	83.81	86.26
Total Conditional AA (mg g^−1^ protein)	473.63	440.47	476.71
BV	62.4	54.9	58.7
EAAI	101.3	88.9	96.8
Requirement index (infants)	86.90	76.28	83.09
Requirement index (preschool child)	94.38	82.85	90.25
Requirement index (schoolchild)	103.27	90.65	98.76
Requirement index (adult)	108.61	95.34	103.86

BAAs, basic AAs; BV, calculated biological value; EAAI, essential AA index.

### 3.6 GC–MS volatile profile analysis of formulated PFHECMs

GC–MS showed that all three formulations shared a complex volatile profile dominated by lipid-derived aldehydes, fatty acids, alcohols, lactones/furanones, and terpenoid hydrocarbons (Table 7). The C6-C10 aldehydes and medium-chain acids, as the most intense peaks, indicated that lipid oxidation and microbial lipolysis are the main drivers of aroma, consistent with reports for fermented and camel milks, where aldehydes, acids, and ketones are key aroma contributors. Across all formulations, hexanal, heptanal, octanal, and nonanal dominated the volatile profile, reflecting oxidation of unsaturated milk and plant lipids and giving green, fatty, and citrus-soapy notes, with minor *α*,*β*-unsaturated aldehydes supporting an overall oxidized-fat aroma typical of fermented milks. Short- and medium-chain acids were also important, with higher hexanoic and heptanoic acid levels and unique presence of pentanoic and hexanoic acids in F1 indicating more intense lipolysis and a stronger sour, cheesy, “goaty” character, whereas F2 and especially F3 showed lower acid levels and therefore a milder, less rancid background that allows fruity and spicy notes to stand out more clearly. Primary and cyclic alcohols increased from F1 to F3, indicating progressive reduction of aldehydes and microbial metabolism that shifts the aroma from sharper oxidized-fat notes to a more rounded fusel–fruity character. Low dimethyl sulfone in F2 and F3 adds slight sulfur complexity, while methylene chloride originates from the extraction solvent and has no sensory relevance. Low-intensity *γ*-dodecalactone and dihydrofuranones across all formulations contribute creamy, coconut, and peach-like notes via hydroxy-fatty acid esterification and Maillard reactions, matching date fruit volatiles (∼8% lactones including *δ*-valerolactone). F3 shows enhanced 2(3H)-furanone derivatives, suggesting greater caramelized, fruity-sweet impact from date–milk–fermentation interactions despite low peak areas, given their low odor thresholds. Monoterpenes and sesquiterpenes (trans-β-ocimene, copaene, caryophyllene, γ-muurolene, calamenene) dominate F3 with lesser amounts in F2 and none in F1, imparting floral, citrus, woody, and spicy notes from nuts/dates/spices (e.g., β-caryophyllene’s clove-pepper). F3’s unique enrichment adds complex woody-spicy top notes, contrasting F1’s dairy-acid/oxidized-fat profile and F2’s intermediate alcohols; all share fermented camel milk’s C6-C10 aldehydes and acids, but F3 offers the most balanced, fruity-spicy, consumer-preferred aroma.

**Table 7:** GC–MS volatile profile analysis of different formulated PFHECMs.

No.	Name	RT	Formula	F1	F2	F3
1.	Methylene chloride	3.428	CH_2_Cl_2_	7.31	9.65	9.65
2.	Pentanoic acid, 4-methyl-	4.379	C_6_H_12_O_2_	5.49	–	–
3.	1-Heptene, 3-methyl-	4.854	C_8_H_16_	–	2.89	1.34
4.	1-Pentanol	4.867	C_5_H_12_O	2.57	–	–
5.	Hexanal	5.223	C_6_H_12_O	18.11	19.02	10.17
6.	1-Hexanol	6.381	C_6_H_14_O	1.6	4.43	6.83
7.	Heptanal	6.913	C_7_H_14_O	9.15	11.51	6.91
8.	trans-.beta.-Ocimene	7.579	C_10_H_16_	–	–	0.77
9.	Pentanoic acid	7.626	C_5_H_10_O_2_	3.9	0.99	–
10.	Dimethyl sulfone	7.813	C_2_H_6_O_2_S	–	0.22	0.19
11.	2-Heptenal, (E)-	8.22	C_7_H_12_O	–	0.37	–
12.	1-Heptanol	8.583	C_7_H_16_O	–	2.29	3.32
13.	Octanal	9.208	C_8_H_16_O	8.07	9.92	6.08
14.	Geranyl propionate	9.86	C_13_H_22_O_2_	–	–	4.23
15.	Hexanoic acid	10.698	C_6_H_12_O_2_	9.53	–	–
16.	Cyclohexanebutanoic acid	10.159	C_10_H_18_O_2_	–	–	3.54
17.	2-Octenal, (E)-	10.703	C_8_H_14_O	–	1.04	1.11
18.	2(3H)-Furanone, dihydro-5-pentyl-	10.897	C_9_H_16_O_2_	–	–	0.61
19.	Cyclohexene,3-propyl-	11.06	C_9_H_16_	1.59	–	–
20.	OCTENAL	11.072	C_8_H_14_O	–	–	4.14
21.	2-Propenoic acid, octyl ester	11.179	C_11_H_20_O_2_	1.76	–	–
22.	Cyclohexanol, 3,5-dimethyl-	11.692	C_8_H_16_O	3.45	2.51	1.91
23.	Nonanal	11.854	C_9_H_18_O	13.53	19.87	14.09
24.	Heptanoic acid	12.618	C_7_H_14_O_2_	6.61	3.04	1.77
25.	2-Nonenal, (E)-	13.374	C_9_H_16_O	2.35	1.3	1.08
26.	2(3H)-Furanone, dihydro-5-propyl-	13.525	C_7_H_12_O_2_	1.01	1.12	0.55
27.	Decanal	14.5	C_10_H_20_O	2.44	3.89	2.76
28.	2-Decenal, (E)-	16.052	C_10_H_18_O	0.43	0.39	0.26
29.	.gamma.-Dodecalactone	16.271	C_12_H_22_O_2_	0.32	0.55	0.37
30.	Cinnamaldehyde, (E)-	16.855	C_9_H_8_O	–	1.64	4.58
31.	Nonanoic acid	16.964	C_9_H_18_O_2_	0.41	–	–
32.	Dodecanoic acid, 3-hydroxy-	17.128	C_12_H_24_O_3_	0.25	–	–
33.	1,5,5-Trimethyl-6-methylene-cyclohexene	18.266	C_10_H_16_	–	0.32	0.61
34.	(1R,2S,6S,7S,8S)-8-Isopropyl-1-methyl-3-methylenetricyclo[4.4.0.02,7]decane-rel-	18.76	C_15_H_24_	–	–	0.14
35.	Copaene	18.898	C_15_H_24_	–	1.58	2.9
36.	2H-Pyran, tetrahydro-2-(12-pentadecynyloxy)-	18.91	C_20_H_36_O_2_	0.12	–	–
37.	Caryophyllene	20.061	C_15_H_24_			0.43
38.	1H-Cyclopropa[a]naphthalene, 1a,2,3,5,6,7,7a,7b-octahydro-1,1,7,7a-tetramethyl-, [1aR-(1a.alpha.,7.alpha.,7a.alpha.,7b.alpha.)]-	20.374	C_15_H_24_	–	–	0.35
39.	Benzene, 1-(1,5-dimethyl-4-hexenyl)-4-methyl-	21.419	C_15_H_22_	–	1.45	3.47
40.	(R)-1-Methyl-4-(6-methylhept-5-en-2-yl)cyclohexa-1,4-diene	21.713	C_15_H_24_	–	–	0.89
41.	Naphthalene, 1,2,4a,5,6,8a-hexahydro-4,7-dimethyl-1-(1-methylethyl)-, (1.alpha.,4a.alpha.,8a.alpha.)-	21.919	C_15_H_24_	–	–	1.47
42.	.gamma.-Muurolene	22.007	C_15_H_24_	–	–	1.38
43.	Naphthalene, 1,2,3,5,6,8a-hexahydro-4,7-dimethyl-1-(1-methylethyl)-, (1S-cis)-	22.401	C_15_H_24_	–	–	1.47
44.	cis-Calamenene	22.513	C_15_H_22_	–	–	0.64

– Not detected.

## 4 Discussion

Fortified camel milk offers compelling nutritional and compositional advantages for both athletes and healthy individuals, including higher protein and electrolyte content and the presence of bioactive components, which may translate into functional benefits; however, direct evidence from controlled intervention studies remains limited (10, 48). For athletes, camel milk’s potent IGF-1-like activity and high protein content supercharge lean muscle growth, slash recovery times, and deliver powerful anti-inflammatory defense against training injuries (49, 50). Meanwhile, date and nut fortification supercharges antioxidant power, turbocharges immunity through bioactive peptides and vitamins, delivers sustained energy without blood sugar spikes, and promotes rock-solid metabolic health, daily vitality, and potent anti-aging effects for everyone (51, 52).

The formulas’ protein content explodes from 3.13 to 9.13% through battle-tested nut mix + KDP fortification, routinely tripling baseline levels while massively amplifying fiber and energy density, just like date-enriched camel milks that super-stabilize probiotics via powerful prebiotic polysaccharides (10, 53). At 9.13 g 100 mL^−1^, the protein content of F3 exceeds typical values reported for plain camel milk and falls within the range of some commercial high-protein dairy beverages, while nut oils contribute to a marked increase in fat and energy content that may improve mouthfeel and caloric density; however, direct sensory or digestibility comparisons with commercial products were not undertaken in this study (25, 54). Fiber gains are expected to synergize with the added probiotics in supporting gut health, and energy density reached 233 kcal 100 mL^−1^ in F3. However, probiotic viability and counts during fermentation and storage were not determined in this study and should be quantified in future work to substantiate the functional status of the product. The camel’s hypoallergenic profile offers superior digestibility compared with cow milk, though bioavailability, culture stress from high solids, fat oxidation, and sensory impacts from viscosity warrant further study (30, 52).

The increase in the TPC validates that KDP and nut mix are rich in bioactive compounds; hence, F2 and F3’s profiles match high antioxidant levels, which may support athletic recovery (54–56). Flavonoid escalation underscores selective extraction of quercetin/kaempferol from dates/nuts, correlating tightly with DPPH-RSA, as in Khalas date studies showing flavonols driving 60–80% RSA (57). Compared to fermented camel milks post-fortification, F3 excels due to non-fermentative preservation of phenolics, though heat processing risks degradation (30). In the broader context, the high phenolic content and DPPH-RSA values observed for F3 are comparable to those reported for some date- and nut-enriched fermented dairy products, which have been associated with reduced oxidative markers *in vivo* (52, 53). These parallels indicate that the formulation may have potential for metabolic and immune support, but direct functional evaluations were beyond the scope of the present study (58, 59).

The L* decline with added nut mix matches patterns in date-fortified camel milk and desserts, where solids like date pulp darken products toward cream-brown hues. Camel milk’s inherent whiteness (L* > 80 from casein/vitamins) drops predictably to ~ 65 in F3 via pigment dilution from KDP/nuts (25, 60). Subtle H° shifts highlight nut browns tempering date golds. ΔE ~ 19–20 signals an obvious visual change from F1, which indicates alignment with phenolic boosts, making F2/F3 desirable for signaling functionality. F2 may balance appeal best, F3 for health-niche intensity (61, 62).

The FA compositions align with camel milk literature, where palmitic (∼18–35%), oleic (∼22–39%), and stearic acids predominate, but SFAs typically comprise 32–66%, higher than bovine milk (∼65–70%) yet with favorable MUFA/PUFA ratios due to smaller fat globules and desert-adapted metabolism (63). F1’s high SFA (65.82%) mirrors raw camel milk (e.g., 66.4% SFA; (63)), but progressive desaturation in F2/F3 reflects nut/date inputs rich in oleic/linoleic acids and probiotic biohydrogenation enhancing C18:1/C18:2. Compared to fermented camel milk studies showing stable palmitic/oleic dominance post-LAB (∼42% C16:0), F3’s 24% PUFA exceeds reports (1–3%; often linoleic <2%), likely from nut lipids, positioning it superior for omega-6 needs (64–66). Critically, the reduction in SFA and increase in PUFA observed in F3 relative to typical camel milk profiles are consistent with more favorable dietary lipid indices and could contribute to improved omega-6 intake; nonetheless, calculation of specific atherogenic or thrombogenic indices and clinical validation were not performed and should be pursued in future studies. However, low linolenic (C18:3, <2.5%) limits omega-3, suggesting flax/enrichment; fermentation minimally altered FAs, unlike cow milk, where LAB boosts CLA/shorts (67, 68), warranting strain optimization. Overall, results validate nut/date/probiotic synergies for functional camel dairy, outperforming standards in unsaturated FA balance (63, 65, 66).

Regarding the fate of AAs, the observed EAA dilution aligns with the inherent imbalance in nut proteins, in which EAAs constitute only 26–27% of TAAs (e.g., cashews 37%, almonds 27%), with NEAAs such as glutamate and arginine dominating (15, 69). This echoes nut-supplemented yogurts where walnut/hazelnut blasts slashed EAA scores but supercharged arginine/proline levels, skyrocketing sensory appeal, health benefits, and digestibility (70). In fermented camel milk contexts, similar shifts occur during shubat fermentation, where lysine remains dominant, but NEAAs such as threonine and alanine increase 1.1–1.7-fold due to microbial proteolysis and substrate effects (71).

Camel milk’s baseline superiority, with EAA/TAA ratios of 0.43–0.52 in whey/casein fractions, exceeds that of cow milk, providing resilience against dilution (12, 72). F3 vs. F2’s dramatic EAA rebound unleashes ferocious non-linear blending dynamics—nut proteins’ stubborn low solubility triggers savage Lactobacillus proteolysis in camel milk, exploding free leucine/lysine levels like a biochemical sledgehammer (13, 73). Unlike plant-based milks (EAA < 25% TAAs), PFHECMs retain >25% EAAs and outperform soy/oat hybrids (16, 74). Critically, the cystine surge addresses camel milk’s sulfur AA limitation (low methionine and cystine), potentially improving PDCAAS scores and glutathione synthesis for antioxidant benefits (75). Arginine enrichment (up 26% in F3) supports cardiovascular health, echoing nuts’ role in inflammation modulation (15). Limitations include unspecified nut types, potentially affecting reproducibility (e.g., peanuts have higher EAAs than almonds), and lack of digestibility assays; future *in vivo* studies could validate bioavailability (76). Overall, these PFHECMs exemplify the valorization of camel milk with nuts, enhancing functional NEAAs while preserving EAA adequacy for hypoallergenic, sustainable nutrition.

The elevated BCAAs align with camel milk’s leucine-rich whey/casein (~10–12% BCAAs of protein), further enhanced relative to total protein in nut blends despite absolute dilution (12, 72). Basic AAs’ recovery in F3 mirrors nut arginine dominance (e.g., almonds/peanuts 10–12% Arg), complementing milk lysine for polyamine/NO synthesis (69, 72). Conditional AAs’ prominence (44–49% TAAs, F3 highest) exceeds that of cow milk yogurt (~40%), attributable to proline-rich camel *β*-casein (65%) and nut glycine/proline, both vital for stress metabolism (13, 77). BV/EAAI values (54–101%) surpass many plant proteins (soy EAAI ~90%, nuts ~70–85%) and approach egg/milk (100%), with nut addition causing ~10% drop but retaining adequacy >FAO minimums (43, 78). Limiting sulfur- and aromatic amino acids is reminiscent of fermented camel milks (shubat, BV ~ 60–70%), where proteolysis boosts Cys but not Met (71). Compared to quinoa-fortified camel yogurt (EAAI 85–95%), nuts better preserve BV via arginine/BCAAs (1). Age indices highlight adult/schoolchild suitability (>58%), limited infant use without SAA fortification. Critically, fermentation likely increases bioavailability via peptides (e.g., Leu-rich peptides from camel caseins), thereby enhancing mTOR/insulinogenic effects (79). Future trials should assess PDCAAS/DIAAS *in vivo*, nut specificity, and proteolysis during shelf-life. These PFHECMs exemplify camel milk valorization, rivaling bovine products in nutritional value while offering allergen-free, bioactive benefits (80).

Camel milk is aldehyde/ketone/alcohol-rich, while *shubat* is ester/acid-dominant (81); current formulations occupy an intermediate position-high aldehydes/acids, low ketones/esters-due to controlled probiotic fermentation and plant matrices, retaining key dairy aldehydes (heptanal, hexanal, octanal, nonanal) for recognizable flavor but requiring oxidation control to prevent storage off-flavors. Fermented dairy studies confirm key odorants like hexanal/octanal/nonanal, medium-chain acids (hexanoic/octanoic), and methyl ketones (2-heptanone/nonanone) from lipid oxidation/β-oxidation/decarboxylation, mirrored here in aldehydes/acids but with fewer ketones possibly due to camel/nut matrices or method effects; LAB fermentation boosts acids (as in F1) while reducing some aldehydes/hydrocarbons (67). Reviews note aldehydes/acids add complexity but drive off-flavors in storage, so high hexanal/heptanal/nonanal levels here necessitate strong oxidative stability/packaging; F3’s lactones/fruity furanones align with balancing creamy/fruity notes against aggressive acids/aldehydes (82). Date powder contributes its characteristic volatiles such as octanal, *δ*/*γ*-valerolactone/undecalactone, esters, alcohols to the products, reinforcing fruity/caramel/creamy notes and enriching complexity via Maillard/caramelization/lipid oxidation with milk components, despite dilution/fermentation lowering relative ester (vs. >20%) and lactone (vs. ∼8%) levels compared to pure dates (83, 84).

Nuts supply terpenes/sesquiterpenes (*α*/β-caryophyllene, copaene, ocimene) that provide nutty/woody/spicy/herbal notes, appearing mainly in F3 (>F2) vs. absent F1, suggesting better plant volatile extraction/retention and β-caryophyllene’s clove-pepper-nutty alignment with the nut-date profile (85, 86). Nuts also add oxidizable unsaturated lipids, yielding hexanal/nonanal/lactones (microbially modified), so F3’s rich terpenoid layer blends with dairy/nut oxidation products for a complex dairy-nutty-fruity-spicy aroma. At the same time, terpene-poor F1 stays simpler/milk-acid focused (81, 87). LAB/probiotic strains vary in producing acids/aldehydes/ketones/alcohols/esters, with traditional yogurt starters yielding broader, acid/unsaturated aldehyde-rich profiles vs. milder probiotics; F1’s acid/alldehyde dominance suggests strong lipolysis/oxidation conditions, while F3’s balanced acids + terpenes indicate different cultures/regimes modulating metabolism (67, 88). Probiotic milks show acids dominating as ketones/hydrocarbons decline (*L. plantarum* boosts hexanal/(E)-2-octenal); current aldehydes fit this pattern, but low esters suggest untapped potential for fruity enhancement to mask oxidized notes (67, 89). GC–MS profiles reveal distinct aromas: F1 shows an acidic/pungent/fermented character with intense green-fatty-rancid notes (hexanal/heptanal/acids), while F3 offers a more complex nutty-woody-spicy-creamy-fruity volatile profile than F1, which is consistent with flavor attributes that have been associated with consumer liking in similar nut- and date-based dairy products; however, no sensory or consumer tests were conducted in the present study to confirm preferences (81, 90). Optimization requires oxidative control (low O₂, antioxidants, low-lipolysis cultures) to limit storage aldehyde buildup, plus ester/lactone enhancement for fruitiness, enabling effective camel/nut/date/probiotic synergy when the acid-aldehyde balance is managed (68, 91).

Collectively, these findings show that probiotic-fermented, high-energy camel milk fortified with KD and nut mix is a nutritionally advanced functional beverage that increases protein, antioxidants, and healthy fats while retaining good amino acid quality and acceptable aroma. The blend of camel milk, probiotics, date prebiotics, and nut-derived oils and phenolics forms a synergistic system beneficial for athletes, people with metabolic concerns, and arid-region populations. Further *in vivo* studies on digestibility, stability, and sensory changes are needed to translate these benefits into evidence-based health claims and optimized products. Obviously, this study is subject to several important limitations. First, no formal sensory analysis, descriptive profiling, or consumer acceptability testing was conducted; therefore, conclusions regarding sensory quality or consumer preference are based solely on instrumental color measurements, volatile profiling, and comparisons with previously reported products. Second, the work focused on freshly prepared beverages, and no shelf-life, storage stability, or packaging studies were performed, which limits the ability to extrapolate the current findings to commercial distribution or extended storage. Third, although probiotic LAB cultures were used for fermentation, no microbial enumeration was performed to assess probiotic viability or survival during storage; thus, the functional labeling of these beverages as “probiotic” is based on the use of established probiotic strains rather than verified viable counts, and future studies should incorporate detailed microbiological analyses. Future research should therefore include comprehensive sensory and consumer evaluations, as well as accelerated and real-time shelf-life studies, to validate the practical applicability of these formulations. The aim statement has been revised to focus on compositional and antioxidant characterization.

## 5 Conclusion

PFHECMs beverages sweetened with Khalas date and fortified nut mixes (F1- F3) demonstrated superior nutritional upgrading, achieving 3-fold protein elevation, 5-fold fat increase with healthier unsaturated profiles (PUFA 24.13% in F3), and potent antioxidants (TPC 506 mg GAE 100 mL^−1^). Amino acid quality remained high (EAAI 88.9–101.3%, BV 54.9–62.4%), with nut-driven enrichment in non-essential amino acids such as arginine and cystine offsetting minor essential amino acid dilution, which may be advantageous for muscle metabolism and cardiometabolic health. However, these potential benefits need to be verified in future human studies. Physicochemical enhancements included desirable color shifts (ΔE ≈ 20) and complex volatiles blending dairy aldehydes/acids with nut-date terpenes/lactones for appealing fruity-nutty-spicy profiles, particularly in F3. The studied formulations exhibit favorable compositional characteristics, including higher protein levels, reduced saturated fat proportions, and elevated antioxidant indices compared with typical camel milk, supporting their development as candidate functional beverages for sustainable nutrition; nevertheless, comparative digestibility, probiotic functionality, and health effects must be confirmed through targeted *in vitro* digestion models and human intervention studies. Future *in vivo* trials and shelf-life optimization are recommended to affirm bioavailability and probiotic viability.

## Data Availability

The original contributions presented in the study are included in the article/supplementary material, further inquiries can be directed to the corresponding author.
